# Rictor Activates Cav 1 Through the Akt Signaling Pathway to Inhibit the Apoptosis of Gastric Cancer Cells

**DOI:** 10.3389/fonc.2021.641453

**Published:** 2021-09-01

**Authors:** Rui-zhen Cao, Li Min, Si Liu, Ru-yue Tian, Hai-yan Jiang, Juan Liu, Lin-lin Shao, Rui Cheng, Sheng-tao Zhu, Shui-long Guo, Peng Li

**Affiliations:** ^1^Department of Gastroenterology, Beijing Friendship Hospital, Capital Medical University, National Clinical Research Center for Digestive Disease, Beijing Digestive Disease Center, Beijing Key Laboratory for Precancerous Lesion of Digestive Disease, Beijing, China; ^2^Department of Gastroenterology, Ordos Central Hospital, National Clinical Research Center for Digestive Disease-Ordos Subcenter, Ordos, China; ^3^Department of Gastroenterology, Beijing University of Chinese Medicine Third Affiliated Hospital, Beijing, China; ^4^Department of Gastroenterology, Shanxi Province Cancer Hospital, Shanxi Medical University, Taiyuan, China

**Keywords:** Rictor, Cav 1, Akt, Apoptosis, Gastric cancer

## Abstract

**Background:**

Rapamycin-insensitive companion of mammalian target of rapamycin (Rictor) protein is a core subunit of mammalian target of rapamycin complex 2, and is associated with cancer progression. However, the biological function of Rictor in cancer, particularly its clinical relevance in gastric cancer (GC) remains largely unknown.

**Methods:**

Rictor expression and its association with clinicopathologic characteristics in GC were analyzed by immunohistochemistry. Effect of Rictor and Caveolin-1 (Cav 1) on GC cells apoptosis was evaluated *via* overexpression experiment *in vitro*. Mechanisms of Rictor and Cav 1 in GC were explored through overexpression and knockdown, by immunofluorescence and western blot analyses.

**Results:**

Rictor was upregulated in GC, and mainly located in the cytoplasm of cancer cells. Moreover, higher Rictor levels were associated with worse prognosis. Rictor could inhibit GC cell apoptosis and promote cell growth *in vitro*. The results of immunofluorescence revealed that Cav 1 localized in GC cell membrane but did not co-localize with Rictor. Further, Rictor regulated apoptosis-related proteins, long non-coding RNAs and also activated cellular signaling, thereby positively regulating Cav 1 expression. This effect was attenuated by the Akt inhibitor ly294002. Cav 1 did not significantly affect the ability of Rictor to inhibit tumor cell apoptosis.

**Conclusions:**

Rictor is upregulated in GC and associated with worse prognosis. It inhibits tumor apoptosis and activates Cav 1 through the Akt signaling pathway to inhibit the apoptosis of GC cells. Rictor is, therefore, a promising prognostic biomarker and possible therapeutic target in GC patients.

## Introduction

Gastric cancer (GC) is one of the most common malignancies worldwide, ranking fifth in incidence and third in mortality (1). East Asia is particularly affected by GC, as indicated by the comparative high mortality (2). GC is the second most common cause of death in China, despite a steady decline in western countries. Owing to high rates of metastasis and recurrence, the five-year overall survival rate for advanced GC is 20% (3). In recent years, with the continued advancements in molecular biology, signaling pathways and targeted therapy have gradually become the focus of GC research, and are expected to provide more effective means for the treatment of GC (4). The most recent advancements in GC research are in the area of non-coding nucleic acids such as long-non-coding RNAs and miRNAs (5–9).

The mammalian target of rapamycin (mTOR) signaling pathway is a key pathway that affects progression of GC. It is often highly activated in GC, and is closely related to clinicopathological characteristics, such as recurrence and metastasis. mTOR exists in the form of mammalian target of rapamycin complex 1 (mTORC1) and mammalian target of rapamycin complex 2 (mTORC2). As one of the core subunits of complex mTORC2, Rictor is the skeleton protein of mTORC2. It is critical for stability and necessary for normal functioning of mTORC2. Rictor is mainly responsible for sensing growth factor concentration, regulating cell proliferation, survival, metabolism and cytoskeletal remodeling. It has been demonstrated through immunohistochemical studies that Rictor expression is increased in association with tumor progression, and that it correlates with poor prognosis of GC patients (10). Current studies have confirmed that Rictor promotes cell growth and proliferation by activating protein kinase B (Akt), promoting cell resistance to apoptosis and promoting angiogenesis (11, 12). Rapamycin has not been as successful as expected in clinical trials. The main reason for this may be the different sensitivities of the two mTOR complexes. mTORC1 is sensitive to treatment with rapamycin. Treatment with rapamycin or its analogues primarily inhibits the mTORC1/S6K pathway and alleviates the negative feedback loop receptor (IGF-1R) from S6K to insulin-like growth factor-1, signaling mTORC2 through the complete pathway leading to Akt activation paradoxically (13). The activation of Akt is concerning because it promotes cell survival and drug resistance, and therefore treatment with an mTORC1 inhibitor might not be beneficial. Inhibition of mTORC2 may eliminate the adverse signaling effects of mTOR inhibitors. Therefore, it is important to further study and characterize the potential therapeutic targets of mTORC2 and explore the associated molecular mechanism in tumors, particularly in GC (14–16).

Caveolin-1 (Cav 1), a membrane protein with a relative molecular weight of 2.1–2.4 ×10^4^, is the main component of caveolae, which is involved in malignant transformation, malignant proliferation, invasion, metastasis and many other biological behaviors of cells. Cav 1 enhances RANKL−induced GC cell migration (17) and also plays a role in epithelial to mesenchymal transition (EMT) impacting the clinicopathological features of GC (18, 19). IGF-I receptor (IGF-IR) localizes in caveolae and tyrosine phosphorylates Cav 1. Cav 1 is involved in the internalization of IGF-IR and directly interacts with IGF-IR and its substrate (20). Cav 1 contributes to anchorage‐independent growth and anoikis resistance of human GC SGC‐7901 cells *via* activation of Src‐dependent EGFR‐ITGB1 signaling (21). The mechanosensitive caveolin-1 activation-induced PI3K/Akt/mTOR signaling pathway promotes cancer motility, invadopodia formation and metastasis *in vivo* (22). This is agreement with the general information on Akt signaling in human cancers, including GC (23–26). Given this information, we hypothesized there may be an interaction in GC between Rictor and Cav 1 that affects the biological behavior of tumor cells.

In this study, we report overexpression of Rictor in GC and its association with worse prognosis. Particularly, we revealed an anti-apoptosis effect of Rictor in GC cells and that Rictor activates Cav 1 through the Akt signaling pathway to inhibit the apoptosis of GC cells.

## Materials and Methods

### Patients and Clinical Data

In total, 92 patients with gastric carcinoma who underwent surgical resection were recruited for this study. Among them, 84 cases without distant metastasis received gastrectomy together with a standard D2 lymph node dissection. The study was approved by the Ethic Committee of Capital Medical University (#66128) The other eight metastatic patients with primary tumor complications, such as obstruction or bleeding, underwent palliative stomach resection. Pathological tumor staging was based on the 7th edition of the Union for International Cancer Control (UICC) TNM staging system. All participants had complete follow-up. The overall survival (OS) time was determined from the date of surgery to the follow-up deadline or date of death. The follow-up deadline was July 2015, and the median follow-up period was 8-9 years (OutDo Biotech Co., Ltd. Shanghai, China).

### Reagents and Antibodies

Primary antibodies used were rabbit monoclonal anti-Rictor (ab70374, Abcam, Cambridge, MA), anti-Cav 1(Rabbit mAb #3276), Akt (pan) (Rabbit mAb #4691, Phospho-Akt (Ser473) (Rabbit mAb #4060) (Cell Signaling Technology, Shanghai, China), Caspase-3 monoclonal antibody (CPP32-4-1-18), Bcl-2 polyclonal antibody (PA5-11379), Bax monoclonal antibody (6A7), actin monoclonal antibody (ACTN05 (C4) and Biotin (ThermoFisher, Shanghai, China). Rictor plasmid (Addgene #1860) and Cav 1 plasmid (Addgene27703) were purchased from Addgene (USA). Rictor-siRNA and Cav 1-siRNA were purchased from GenePharma Co., Ltd. (Shanghai, China). Ly294002 (#9901) was purchased from Cell Signaling Technology. Human GC cell lines SGC-7901 and AGS were purchased from iCell Bioscience Inc (Shanghai, China) in Feb 2018.

### Immunohistochemistry

Tissue microarrays were constructed by Shanghai Xinchao Biotechnology Co., Ltd. (Shanghai, China). Prepared slides were incubated at 65°C for 1 h, After incubation, the sections were deparaffinized in xylene and rehydrated in alcohol. Following antigen retrieval with high pressure, endogenous peroxidase activity was blocked with 3% H_2_O_2_ for 20 min. Sections were blocked with goat serum for 1 h and incubated with primary antibody against Rictor (Abcam, dilution of 1:100) overnight at 4°C. The next day, the tissues were incubated with Universal second antibodies (goat anti-rabbit and mouse) for 60 min at room temperature. Immunostaining was carried out with DAB substrate kit (Thermo Scientific, Waltham, USA), followed by immersion into hematoxylin for nuclear counterstaining.

### Scoring of Staining

The results of immunohistochemical staining were evaluated by two independent investigators according to a semiquantitative grading system based on both proportion of stained cells and their intensity. The extent of staining was scored as no staining = 0; <1/3 staining = 1; 1/3 to 2/3 staining = 2; and >2/3 staining =3. Staining intensity was scored as: none = 0; weak =1; medium =2; and strong = 3. The intensity and percentage scores were added to give a final score ranging from 0 to 6. The results of immunostaining were divided into two groups where 0-2 was considered negative (-) and 3-6 was considered positive (+).

### Cell Culture and Plasmid Transfections

The two cell lines, SGC-7901 and AGS were respectively cultured in Dulbecco’s Modified Eagles Medium (DMEM) with 10% Fetal Bovine Serum(FBS) and Ham's F-12K(Kaighn's) medium (F-12K) with 20% FBS in a humidified incubator at 37°C with 5% CO_2_. Cell lines used in the experiments were authenticated using short tandem repeat (STR) profiling in the Genomics core facility of Capital Medical University on an annual basis, with last authentication in April, 2020, and passaged less than 5 times at any given time. When SGC-7901 and AGS cells grew to 50-60% confluency, the Rictor and Cav 1 plasmids and vectors were used to infect cells using lipofectamine 3000 and P3000 (Life Technologies, China), according to the manufacturer’s instructions. Rictor-siRNA and Cav 1-siRNA were transfected into two the cell lines by using lipofectamine 3000 according to the provided protocols. The transfection efficacy was determined by western blot.

### Cell Apoptosis Assay

Cell apoptosis was measured by Annexin V-FITC/PI Apoptosis Detection Kit (KeyGEN, Guangzhou, China). Cells were collected, were digested and isolated in Dulbecco’s Phosphate-Buffered Saline (DPBS), washed with cold phosphate-buffered saline (PBS) 3 times, PBS solution and re-suspended in binding buffer. Cells were stained by AnnexinV-FITC and 7-aminoactinomycinD (7-AAD) (BD Biosciences, New York, USA) for 15min, sorted using the FACS Calibur system (BD Biosciences) and counted apoptotic cells when AnnexinV staining was positive.

### Cell Survival Assay (MTS)

To examine the effects of Rictor on the proliferation of GC cells, one-step 3-(4,5-dimethylthiazol-2-yl)-5-(3-carboxymethoxyphenyl)-2-(4-sulfophenyl)-2H-tetrazolium (MTS) assays were conducted. In total, 2000 cells/well in100μL of medium were seeded in a 96-well plate after transfection, and detected at 8h, 32h, and 56h using an enzyme-labelled meter (Spectramax M3, Molecular Devices, Shanghai, China) 2h after the addition of MTS.

### EdU Cell Proliferation Assay

After Rictor plasmid transfection, the cells were cultivated for 48 h.Then 20,000 cells/well were seeded in a 24-well plate. In total, 100 μL incubation EdU medium(1000:1) was added to each well and incubated for 2 h. After washing with PBS, the cells were fixed for 30 min in 4% paraformaldehyde. Then, 2mg/mL glycine was added to the wells and further incubated for 5 min. Then, the wells were washed with PBS before adding penetrating agent (PBS containing 0.5% Triton X-100) followed by incubation for 10 min, at room temperature in the dark. Further, cells were incubated with Apolle dye for 30 min, penetrant decolorizing cleaned cells for 10 min thrice, Then, for DNA staining, cells were incubated with the reaction solution for 30min in the dark at room temperature. After washing with PBS thrice, the product was tested on Olympus IX51.

### Immunofluorescence

To determine the cellular localization of Rictor and Cav 1, SGC-7901 and AGS cell lines were seeded on sterile coverslips in the well of 6-well plates, washed with PBS three times, then cells were fixed with 4% paraformaldehyde for 15min, permeabilized with 0.25% Triton X-100 in PBS for 10 min, followed by blocking in 5% BSA in PBST for 1h. Cells were then incubated overnight in 4°C with special primary antibody: anti-Rictor (dilution 1:100) and anti-Cav 1(dilution 1:100). The next day, primary antibody was removed, cells were washed with PBS, and incubated in a mixture of two fluorescent secondary antibodies (Alexa Fluor 488–conjugated anti-mouse IgG and Alexa Fluor 594–conjugated anti-rabbit IgG) (dilution1:100,Life Technologies) in the dark for 2h, Cells were stained with DAPI and photographed by confocal microscopy (IX83, FLUOVIEW FV1200, Olympus).

### Western Blot

Treated cells were collected and lysed using lysate buffer on ice for 30 min, and protein concentration was determined by BCA assay. Equal amounts of proteins (30μg) were separated by SDS-PAGE and transferred to nitrocellulose membranes. After blocking in 5% non-fat milk for 2 h, the membranes were incubated with primary antibodies against Rictor, p-Akt (Ser473), Akt, Cav 1, Caspase-3, Bcl-2, Bax or β-actin overnight at 4°C. The following day, membranes were incubated with anti-rabbit or anti-mouse secondary antibodies at room temperature for 1 h. Finally, immunoblots were visualized using enhanced chemiluminescence (ECL) reagent (Thermo Scientific, USA). β-actin was used as a loading control.

### Statistical Analysis

Data are presented as Means ± SD. All statistical analyses were plotted with the GraphPad Prism (Version 8.0.1) and IBM SPSS Statistical 25. Statistical tests are one-sided or two-sided, t tests were conducted to evaluate the differences between two groups, while ANOVA tests were used in multiple comparable groups. Log-rank tests and Kaplan-Meier plots were applied to assess and show differences in overall survival (OS) between subgroups. Cox proportional hazard models were used for multiple-variants analysis. P < 0.05 was considered to indicate a statistically significant difference.

## Results

### Characterization of Rictor Expression

**Figure 1:**
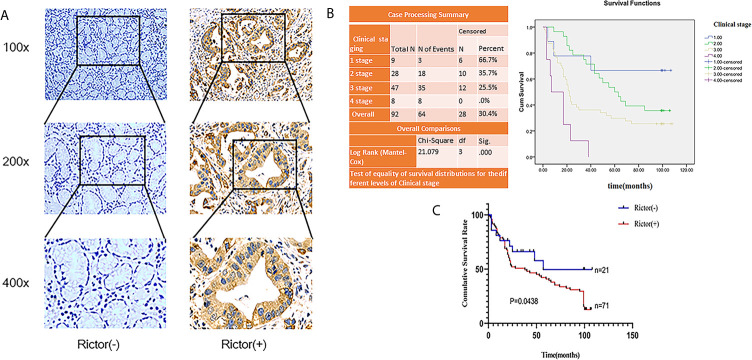
Rictor is upregulated inside GC cells and correlated with poor prognosis in different GC cohorts. **(A)** Representative immunohistochemical stains for Rictor. **(B)** Kaplan-Meier survival analysis with log-rank test for OS in all 92 patients with gastric cancer sorted by immunostaining of Rictor. **(C)** Survival analysis of 92 gastric cancer cases.

To evaluate the expression of Rictor in GC, immunohistochemical assays were performed on 92 patients with GC and adjacent normal tissues. We found that Rictor localized in the cytoplasm of cancer cells, but not in the surrounding stroma cells. We also found that expression of Rictor was significantly increased in GC tissues compared with normal tissues (**Figure 1A**). Kaplan-Meier survival analysis with log-rank test for OS in all 92 patients with gastric cancer (**Figure 1B**) and survival analysis of 92 cases in gastric cancer (**Figure 1C**). Clinicopathological statistical analyses indicated that Rictor expression was correlated with tumor size, depth of invasion, lymph node metastasis, TNM stage, WHO grading and tumor thrombus. There was no significant association with gender, age, tumor location or distant metastasis (**Table 1**). Taken together, our data indicates that Rictor was located in GC cells but not in the surrounding stroma cells in GC tissues, and could be used as a potential prognostic biomarker for GC patients.

**Table 1:** Correlation of Rictor expression with clinicopathological characteristics in 92 gastric cancer patients.

Factors	Cases	Rictor	
		n (%)	P value
Gender			
Male	57	44(81.5)	0.238
Female	35	27(77.1)	
Age(yr)			
≤60	30	22(68.8)	0.470
>60	62	49(79.0)	
Location			
Upper	11	10(90.9)	0.173
Central	24	18(75.0)	
Lower	49	36(73.5)	
Diffuse	8	7(87.5)	
Size			
<5cm	41	35(85.4)	0.019*
≥5cm	51	35(68.3)	
Depth of invasion			
TI+T2	14	13(92.9)	0.026*
T3+T4	78	58(74.4)	
Lymph node metastasis			
Negative	23	18(78.2)	0.037*
Positive	69	53(76.8)	
Distant metastasis			
M0	84	64(76.2)	0.033*
M1	8	7(87.5)	
TNM stage			
I	9	9(100)	0.009*
II	28	20(71.4)	
III	47	35(74.5)	
IV	8	7(87.5)	
Who grading			
Grade 1	14	14(100)	0.029*
Grade 2	68	50(73.5)	
Grade 3	10	7(70.0)	
Tumor thrombus			
Negative	79	60(75.9)	0.046*
Positive	13	11(84.6)	

Statistical analysis indicated that Rictor expression was correlated with tumor size, depth of invasion, lymph node metastasis, TNM stage, WHO grading and tumor thrombus. There was no significant association with gender, age, tumor location and distant metastasis. *P < 0.05.

### Rictor Promoted Growth of GC Cells *In Vitro*

**Figure 2:**
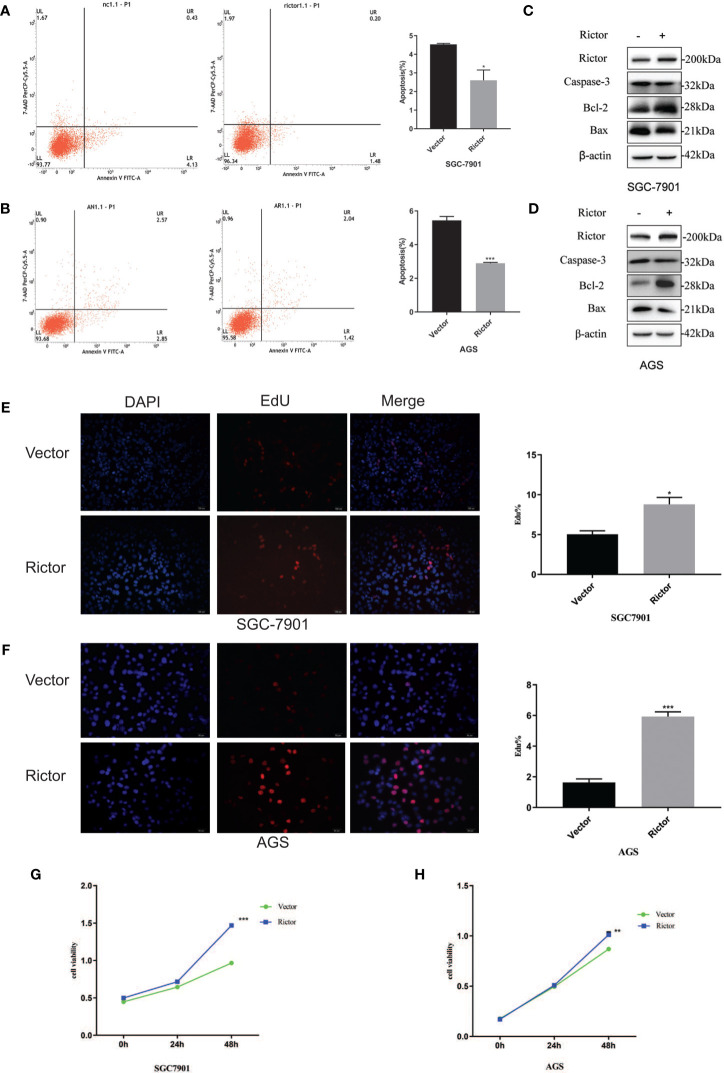
Growth promoting effect of Rictor in gastric cancer cell lines. **(A)** Transfection of Rictor plasmid can inhibit the apoptosis of SGC-7901 cells (n=9). **(B)** Transfection of Rictor plasmid can inhibit the apoptosis of AGS cells (n=9). **(C)** Expression of apoptosis related proteins after transfection with Rictor plasmid in SGC-7901 cells. **(D)** Expression of apoptosis related proteins after transfection with Rictor plasmid in AGS cells. β-actin served as loading control. **(E)** EdU verification that transfection of the Rictor plasmid increased proliferation of SGC-7901 cells (n=3). **(F)** EdU verification that transfection of the Rictor plasmid increased proliferation of AGS cells (n=3). **(G)** Transfection of Rictor plasmid can increase the proliferation of SGC-7901 cells (n=3). **(H)** Transfection of Rictor plasmid can increase the proliferation of AGS cells (n=3). Values represent the Means ± SD. *P < 0.05, **P < 0.01 and ***P < 0.001 as calculated using the Student’s t-test.

**Figure 3:**
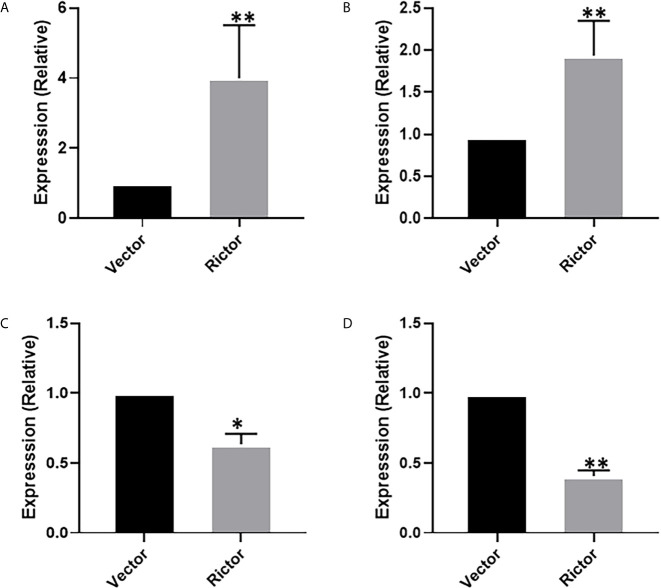
Long-non coding RNAs modulating effect of Rictor in gastric cancer cell line SGC-7901. Transfection of Rictor plasmid increased the expression of lncRNAs **(A)** MALAT-1 and **(B)** GMAN and decreased the expression of lncRNAs **(C)** MEG3 and **(D)** GAS5. Values in control (vector-transfected cells were assigned a value of ‘1’ and those in Rictor-transfected cells are presented as comparative fold-change. *P < 0.05 and **P < 0.01, as calculated using the Student’s t-test.

To investigate the growth-promoting function of Rictor on GC cells, we tested apoptosis rates of SGC-7901 and AGS cells after Rictor overexpression by flow cytometry. The results showed that overexpression of Rictor level significantly inhibited apoptosis in GC cells (**Figures 2A, B**). Rictor down-regulated Caspase-3 and Bax, and up-regulated Bcl-2 to achieve apoptosis-inhibiting effect on GC cells (**Figures 2C, D**). To further explore the biologic functions of Rictor in GC, we overexpressed Rictor in SGC-7901 and AGS cells to test cell viability by EdU (**Figures 2E, F**) and MTS assays (**Figures 2G, H**). The results suggested that overexpression of Rictor markedly promoted cell viability in SGC-7901 and AGS. We further, evaluated effect of Rictor on long-non-coding RNAs and found that Rictor induced MALAT-1 as well as GMAN (**Figures 3A, B**), both of which promote GC tumorigenesis (27, 28). On the contrary, Rictor down-regulated MEG3 and GAS5 (**Figures 3C, D**), both of which are tumor suppressive lncRNAs (29).

### Cav 1 Inhibited Apoptosis of Gastric Cancer Cells

**Figure 4:**
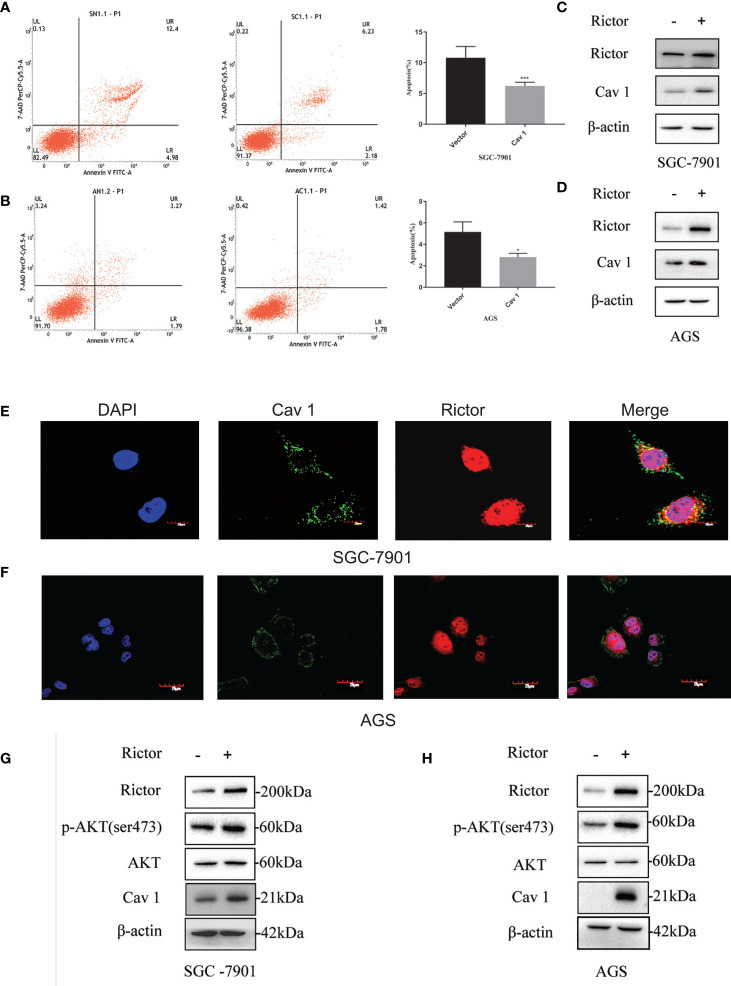
Cav 1-induced inhibition of apoptosis of gastric cancer cells and association between Rictor and Cav 1. **(A)** Transfected Cav 1 plasmid could inhibit the apoptosis of SGC-7901 cells (n=9). **(B)** Transfected Cav 1 plasmid could inhibit the apoptosis of AGS cells (n=9). Values represent the Means ± SD, *P < 0.05 and ***P < 0.001 were calculated using Student’s t-test. **(C)** Western blot analysis of the increase of Cav 1 expression with Rictor overexpression in SGC-7901 cells. **(D)** Western blot analysis of the increase of Cav 1 expression with Rictor overexpression in AGS cells. **(E, F)** Immunofluorescence showed no co-localization between Rictor and Cav 1. **(G)** Western blot analysis of p-Akt and Cav 1 levels increased after transfection of SGC-7901 cells with Rictor plasmid. **(H)** Western blot analysis of p-Akt and Cav 1 levels increased after transfection of AGS cells with Rictor plasmid. β-actin served as a loading control.

To investigate the function of Cav 1 on GC cells, we tested apoptosis rates of SGC-7901 and AGS cells overexpressing Cav 1, by flow cytometry. The results showed that overexpression of Cav 1 level significantly inhibited apoptosis in GC cells (**Figures 4A, B**). After Rictor overexpression in SGC-7901 and AGS cells, western blot analyses showed an increase of Cav 1 expression (**Figures 4C, D**). In GC cells, Rictor and Cav 1 did not co-localize (**Figures 4E, F**), suggesting there was no direct interaction between them. Western blot analysis indicated that p-Akt and Cav 1 levels increased after transfection of both SGC-7901 and AGS cells with the Rictor plasmid (**Figures 4G, H**).

### Rictor Activates Cav 1 Through the Akt Signaling Pathway to Inhibit Apoptosis of Gastric Cancer Cells

**Figure 5:**
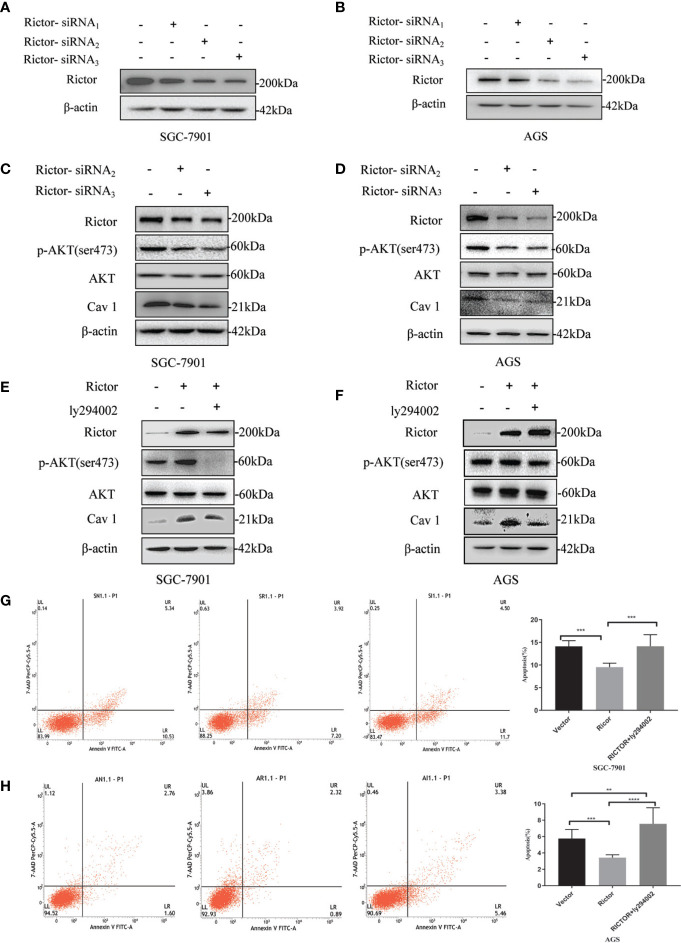
Association between Rictor and Cav 1 analyzed using western blot and apoptosis detection. **(A, B)** Two different cell lines were transfected with Rictor-siRNA respectively to verify Rictor-siRNA knock down efficiency. **(C)** Protein levels of p-Akt and Cav 1 induced by Rictor knockdown in SGC-7901 cells were assessed. **(D)** Protein levels of p-Akt and Cav 1 induced by Rictor knockdown in AGS cells. **(E)** After transfection of Rictor plasmid in SGC-7901 cells for 24 h and addition of 20 μM ly294002 for 6 h, the changes of p-Akt and Cav 1 protein levels were detected by western blot. **(F)** After transfection of Rictor plasmid into AGS cells for 24 h, and addition of 20 μM ly294002 for 6 h, the changes of p-Akt and Cav 1 protein levels were detected by western blot. β-actin served as loading control. **(G)** Apoptosis of SGC-7901 cells after 24 h transfection with Rictor plasmid and 24 h treatment with 20 μM ly294002 (n=3). **(H)** Apoptosis of AGS cells after 24 h transfection with Rictor plasmid and 24 h treatment with 20 μM ly294002 (n=3). Values represent the Means ± SD. **P < 0.01, ***P < 0.001 and ****P < 0.0001 were calculated using Student’s t-test.

Two different cell lines (SGC-7901 and AGS) were transfected with Rictor-siRNA to verify Rictor-siRNA knock down efficiency (**Figures 5A, B**). After transfection with Rictor-siRNA, the protein levels of p-Akt and Cav 1 were analyzed (**Figures 5C, D**). After transfection of Rictor plasmid into SGC-7901 and AGS cells for 24 h, 20μM of the Akt inhibitor ly294002 was added. Western blot analysis showed the levels of p-Akt and Cav 1 decreased (**Figures 5E, F**). Apoptosis increased in both cell lines, after transfection with the Rictor plasmid for 24 h and a 24 h treatment with Akt inhibitor ly294002 (**Figures 5G, H**).

### Knockdown Cav 1 Had No Effect on Apoptosis Inhibition by Rictor Overexpression

**Figure 6:**
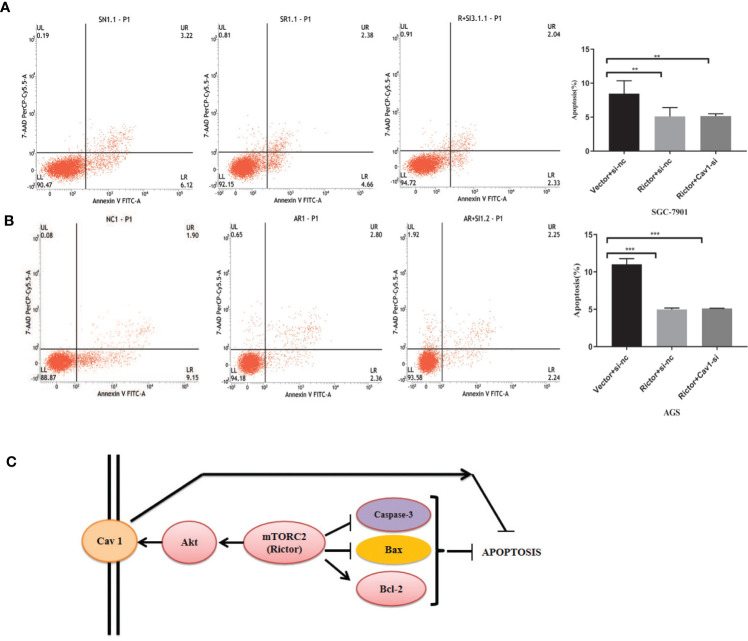
Cav 1 knockdown has no effect on apoptosis inhibition induced by Rictor overexpression. **(A)** Co-transfection of Rictor plasmid and Cav 1-siRNA in SGC-7901 cells indicated that knocking down Cav 1 did not affect the inhibition of Rictor on apoptosis (n=3). **(B)** Co-transfection with Rictor plasmid and Cav 1-siRNA in AGS cells indicated that knocking down Cav 1 did not affect the inhibition of Rictor on apoptosis (n=3). Values represent the Means ± SD. *P < 0.05, **P < 0.01 and ***P < 0.001 were calculated using Student’s t-test. **(C)** Signaling pathway of Rictor and Cav 1.

To investigate whether Cav 1 had a direct effect on apoptosis inhibition due to Rictor overexpression, we co-transfected the Rictor plasmid and three Cav 1-siRNAs into SGC-7901 and AGS cells, then tested apoptosis rates by flow cytometry. The results indicated that knocking down Cav 1 had no effect on apoptosis inhibition by Rictor overexpression (**Figures 6A, B**).

Finally, the prospective Rictor signaling pathway is summarized in **Figure 6C**. Rictor inhibited the apoptosis of tumor cells by regulating apoptosis-related proteins, and activated the tumor cell apotosis inhibition effect of Cav 1 through the Akt signaling pathway. Cav 1 did not directly affect the inhibitory effect of Rictor on apoptosis.

## Discussion

Oncogenic signaling and metabolic alterations are interrelated in cancer cells. mTOR, which is frequently activated in cancer, controls cell growth and metabolism (30). This signaling pathway is often highly activated in GC, and is closely related to clinicopathological characteristics, such as recurrence and metastasis (31). Studies have proved, through immunohistochemistry, that the increase in Rictor expression is associated with tumor progression and poor prognosis in GC patients (10). It has also been reported that p-mTOR could be used as a prognostic marker, suggesting that investigations of mTOR inhibitors may provide a novel therapeutic approach. mTOR exerts additional functions when combined with Rictor to form mTORC2 (32). However, the expression and role of Rictor remains unclear in GC. In this study, we found that Rictor was expressed at 77.17% in GC versus 25.33% in adjacent mucosa, and this overexpression significantly correlated with tumor size, depth of invasion, lymph node metastasis, and TNM stage. This indicates that Rictor is involved in tumor growth and metastasis. In addition, Kaplan-Meier analysis showed that Rictor positive expression predicted poorer overall survival. In renal cancer, Rictor is closely related to metastasis and cell proliferation of renal cancer cells, and the downregulation of Rictor could inhibit metastasis and proliferation, thus inhibiting tumor growth (33). In mouse mammary glands, downregulation of Rictor can block the expansion and obstruction of ductal branches regulated by mTORC2, as well as the invasion and survival of mammary epithelial cells (34). Rictor is highly expressed in human glioblastomas, and activation of mTORC2 also enhances phosphorylation of the downstream substrate Akt (35). Our results are consistent with the majority of the reported findings. We propose that Rictor positive expression is implicated in progression and metastasis of GC, and might serve as a novel biomarker and therapeutic target. The present study of Rictor expression by immunohistochemistry in human cancer tissues suggests that targeting Rictor/mTORC2 may attenuate tumor growth. As a result, we found that Rictor overexpression can affect the expression of apoptosis-related proteins such as Caspase-3, Bax, and Bcl-2, thereby reducing the apoptosis of GC cells. Targeted inhibition of Rictor leads to growth inhibition and induces apoptosis in both rapamycin-sensitive and rapamycin-resistant CRCs, suggesting that selective targeting of mTORC2 may represent a novel therapeutic strategy for treatment of CRC (36). The effects of Rictor on cell proliferation and apoptosis have been observed in malignant pheochromocytoma (37), melanoma (38, 39) and lung cancer (40).

Resistance of solid tumors to chemo-and radiotherapy remains a major obstacle in anti-cancer treatment (41). Cav 1 has gained attention owing to its high expression in many tumors, and high Cav 1 levels are correlated with a worse clinical outcome. Cav 1 plays an important role in modulating tumor host interactions by promoting tumor growth, metastasis, therapy resistance, and cell survival. Understanding these interactions and thus, inhibiting Cav 1, may offer a novel strategy for preventing cancer therapy resistance and improving clinical outcomes. Cav 1 is an integral membrane protein that is abundantly expressed in adipocytes, endothelial cell, pneumocytes, fibroblasts, and muscle cells (42–44), and is involved in cell signaling and transport. It is also involved in caveola-mediated endocytosis, and therefore regulates numerous cellular processes by transmitting extracellular signals *via* intracellular pathways (45, 46). Cav 1-dependent signal transduction regulates cell cycle, proliferation and invasion (47) and cell death (48–50). The molecular mechanisms of Cav 1, mediating radio and chemoresistance of cancer cells, have been increasingly studied in the last few years. High Cav 1 expression, correlated with worse clinical outcomes and drug resistance, has been reported in ovarian, colon, and breast cancer (51–53). High Cav 1 expression is also associated with RAF-ERK signaling, cell cycle progression and colony forming ability (54). Patients with Cav 1-positive tumors, post-gastrectomy, display decreased disease-free and overall survival (55). Moreover, Cav 1 expression is associated with poor prognosis in GC (56). Cav 1 expression is low in GC patients in comparison to healthy stomach tissue. Additionally, GC cell lines of primary tumors display low levels of Cav 1, whereas cell lines originated from metastases show high expression levels (57). In human SGC-7901 cells, Cav 1 promotes anchoring‐independent growth and apoptosis resistance by activating Src-dependent EGFR-ITGB1 signaling, which may indicate Cav 1 to be a potential therapeutic target for gastric metastasis (21). Mechanically sensitive caveolin-1 activation induces the PI3K/Akt/mTOR signaling pathway to promote motility and invasive *in vivo* formation, and metastasis of breast cancer (22).

We found that overexpression of Rictor led to increased expression of Cav 1. Similarly, knocking down Rictor led to decreased expression of Cav 1. By immunofluorescence, Rictor was located in the cytoplasm and Cav 1 was located on the cell membrane. There was no co-location between Rictor and Cav 1. We speculate that there is no direct interaction between them, and that Rictor regulates Cav 1 through the Akt signaling pathway by activating Akt Ser473. This regulatory effect was reduced when Akt was inhibited by the PI3K/Akt inhibitor ly294002, and apoptosis of GC cells also increased significantly. However, siRNA knockdown of Cav 1 did not affect the apoptotic resistance of Rictor to GC cells. We speculate that Rictor is upstream of Cav 1 and has a positive regulatory effect on Cav 1; however, Cav 1 does not have a significant effect on Rictor. These results indicate that inhibition of Rictor/mTORC2 may prevent undesired oncogenic effects of Cav 1 simultaneously.

Targeting Rictor/mTORC2 as an anticancer therapy is an attractive prospect, since 68% of GC patients show elevated Akt levels, and mTORC2 is a critical kinase to phosphorylate Ser473 residue for full activation of Akt. Rictor/mTORC2 might be more deleterious to cancer cells than to normal cells, leading to less toxicity by selective mTORC2 inhibition. Our results also support the hypothesis that Rictor plays a critical role in GC proliferation. Our findings provide the rationale for further investigations toward mTOR kinase inhibitor targeting both mTOR complexes or specifically targeting mTORC2 as an effective therapeutic candidate against GC in the future (58).

In summary, the current study provides substantial new evidence that Rictor is involved in GC cell proliferation and increases the tumor-promoting effect of Cav 1, indicating that Rictor may serve as a feasible therapeutic target for GC.

## Data Availability Statement

The original contributions presented in the study are included in the article/supplementary material. Further inquiries can be directed to the corresponding authors.

## Ethics Statement

The studies involving human participants were reviewed and approved by Ethic Committee of Capital Medical University. The patients/participants provided their written informed consent to participate in this study.

## Author Contributions

All authors contributed to the article and approved the submitted version. R-zC, LM, SL, and R-yT performed experiments and carried out the data analysis. H-yJ, JL, L-lS, and RC helped perform experiments and collect the primary data. S-tZ and L-lS helped analyze data. S-lG and PL conceived and designed the study. R-zC, LM, S-lG, and PL drafted the manuscript.

## Conflict of Interest

The authors declare that the research was conducted in the absence of any commercial or financial relationships that could be construed as a potential conflict of interest.
